# Supplementary material for: Zebularine potentiates anti-tumor immunity by inducing tumor immunogenicity and improving antigen processing through cGAS-STING pathway
Source: Commun Biol. 2024 May 16;7:587. doi: 10.1038/s42003-024-06271-w (PMC11099016; doi:10.1038/s42003-024-06271-w)
Supplement: Supplementary file 2 — Supplementary information [file 42003_2024_6271_MOESM2_ESM.pdf]

Supplementary Information for

Zebularine potentiates anti-tumor immunity by inducing tumor immunogenicity and improving antigen processing through cGAS-STING pathway

Yong Zhang[1,2], Heng Zhao[1,2], Weili Deng[1,2], Junzhong Lai[3], Kai Sang[1,2], Qi Chen[1,2*]

1. Fujian Key Laboratory of Innate Immune Biology, Biomedical Research Center of South China, Fujian Normal University Qishan Campus, Fuzhou, Fujian Province 350117, China.

2. College of Life Science, Fujian Normal University Qishan Campus, Fuzhou, Fujian Province 350117, China.

3. The Cancer Center, Union Hospital, Fujian Medical University, Fuzhou, Fujian Province 350117, China.

* Correspondence author: Qi Chen, E-mail: chenqi@fjnu.edu.cn.

This file contains:

- \- Supplementary Figures 1 to 11
- \- Supplementary Figure Legends
- \- Uncropped blots for all the Western blots of the paper
- \- Supplementary Table 1 to 2 （Includes reagent information and primer sequence）

Supplementary Figure 1

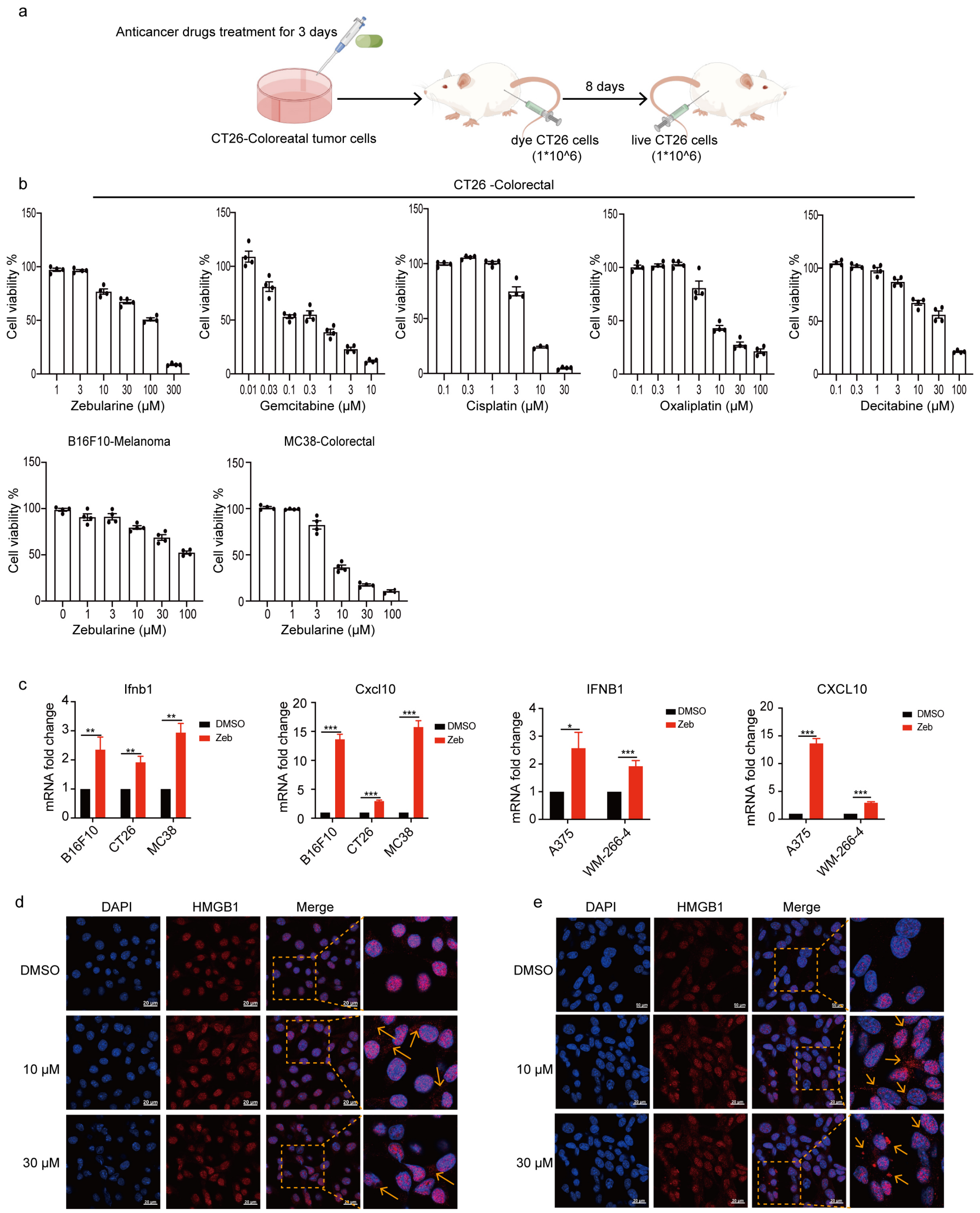

Supplementary Figure 1. Zebularine-induced immunogenic cell death in tumor cells.

a Schematic of vaccination experiments (By Figdraw, ID: PUTIT6c969). CT26 tumor cells were pre-treated with zebularine (zeb, 150 μM), decitabin (100 μM), oxaliplatin (100 μM),

platinum (30 μM), or gemcitabine (10 μM) for 3 days followed by subcutaneous inoculation into BALB/c mice as a vaccine, freeze-thawed as negative control. After 8 days, mice were re-challenged with live CT26 cells, and live CT26 cells were also implanted into non-immunized mice as a control.

b CT26 cells were treated with different concentrations of zebularine, gemcitabine, cisplatin, oxaliplatin, or decitabine for 72 hours, and cell proliferation was detected by Cell Counting Kit-8 (CCK8) assay. B16F10, MC38, and CT26 cells were treated with different concentrations of zebularine for 72 hours, and cell proliferation was detected by Cell Counting Kit-8 (CCK8) assay.

c mRNA levels of indicated genes from B16F10, CT26, MC38, A375, and WM-266-4 cells treated with zebularine were analyzed using RT-qPCR.

d,e The leakage of HMGB1 from the nucleus to the cytoplasm after treatment of B16F10 (d) and CT26 (e) cells with different concentrations of zebularine for 72 h, observed by confocal microscopy.

Data are presented as mean ± SEM of at least three independent experiments. *p<0.05; **p<0.01; ***p<0.001 by Student's t test.

Supplementary Figure 2

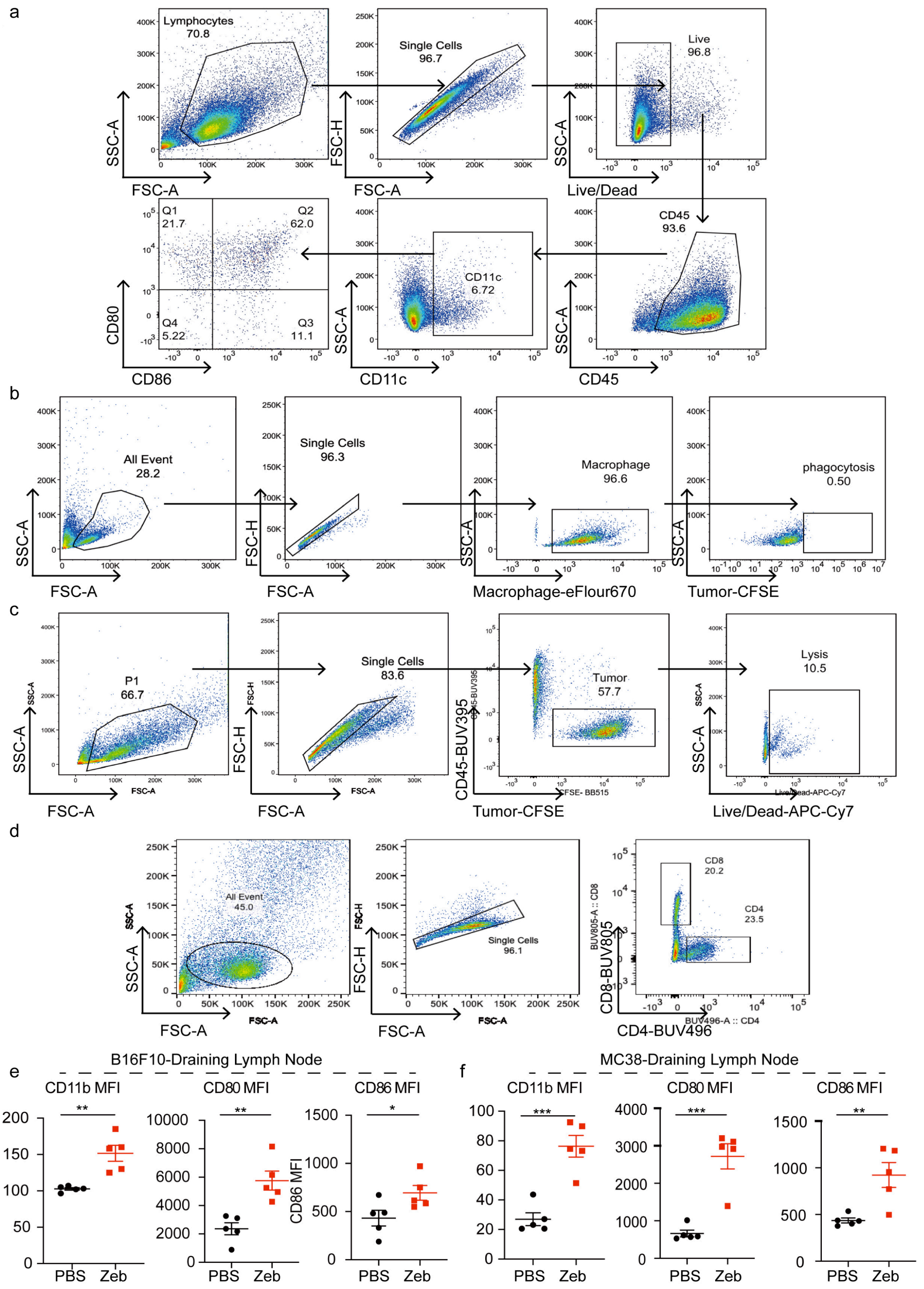

Supplementary Figure 2. Gating strategies for flow cytometry experiments.

a Gating strategy used for DC maturation after pretreatment with LPS (1 mg/mL) for 48 h.

These data correspond to data shown in Figures 2c-d. Flow cytometry analysis of the co-expression of $CD80^+$ and $CD86^+$ on DCs after co-incubation with pre-treated tumor cells.

b Gating strategy used for phagocytosis. Gating strategy to identify fraction of BMDM actively phagocytosing tumor cells following co-culture in response to drug treatment. Gating strategy is the same as that for BMDC phagocytosis.

c Gating strategy for assessing lymphocyte cell killing in vitro.

d Gating strategy for assessing Naive T cell differentiation in vitro.

e,f Expression (MFI) of $CD11b^+$, $CD80^+$, and $CD86^+$ on cells in draining lymph nodes (dLNs) of tumor-bearing mice pre-treated with zebularine, detected by flow cytometry.

Data are presented as mean ± SEM of at least three independent experiments. *$p<0.05$; **$p<0.01$; ***$p<0.001$ by Student's t test.

Supplementary Figure 3

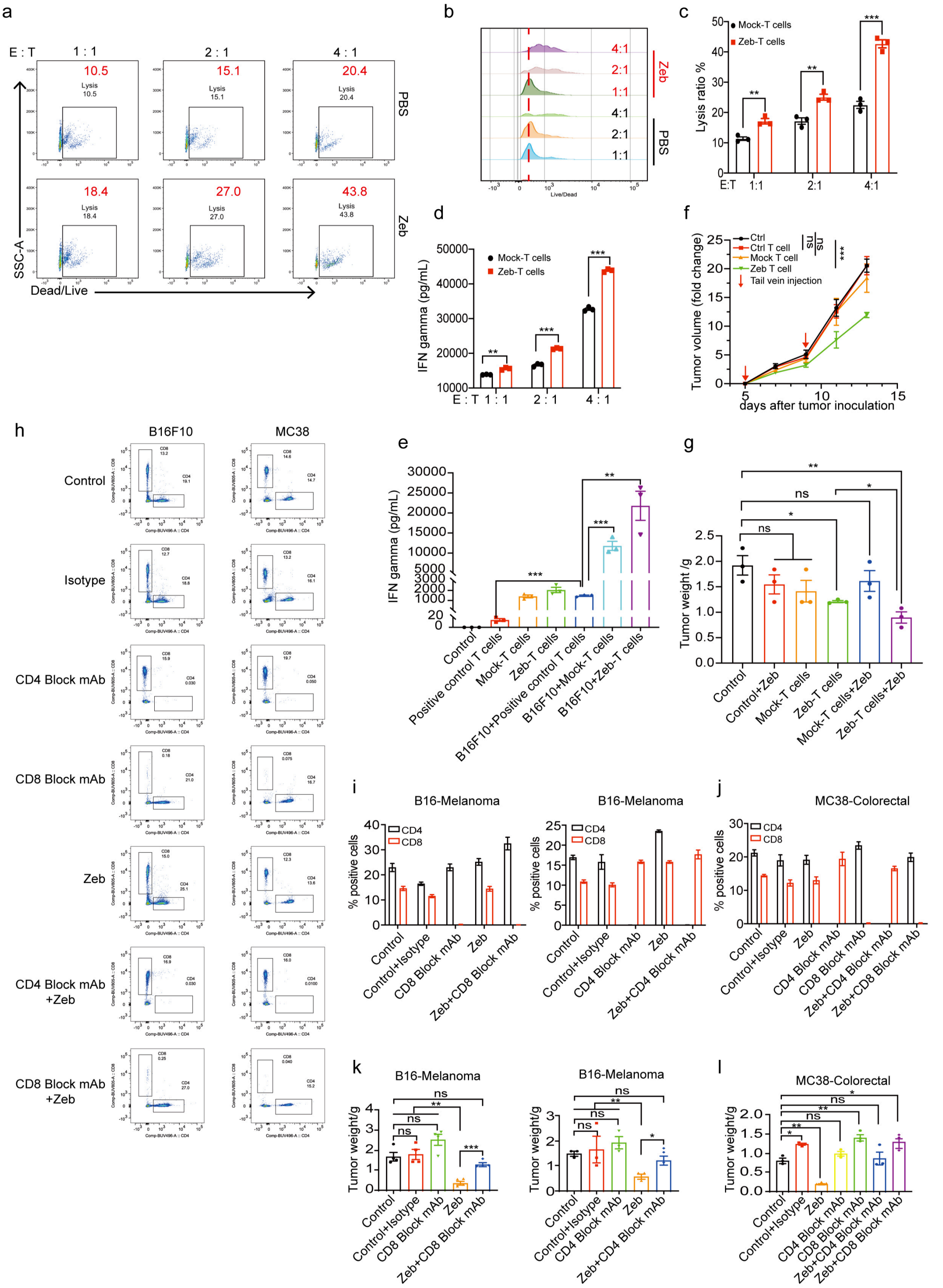

Supplementary Figure 3. Zebularine affects tumor growth in a T cell-dependent manner.

a-c Untreated and zebularine-treated mouse spleen T cells co-cultured with B16F10 cells

at different proportions of Effector to Target (1:1, 2:1, 4:1) for 24 h. Relative killing was calculated by analyzing the remaining tumor cells ($CFSE^+$, $Fvs780^+$) by flow cytometry (gating strategy shown in Figure S2c).

d-e Interferon-γ (IFN-γ) release measured by ELISA.

f Tumor growth of B16F10 tumor-bearing mice.

g Tumor weight after subcutaneous injection of B16F10 cells into NCG mice.

h-j Percentage of $CD4^+$ or $CD8^+$ cells detected by flow cytometry. These data correspond to data shown in Fig. 3c-e.

k-l Tumor weight corresponding to data shown in Fig. 3c-e.

Data are presented as mean ± SEM of at least three independent experiments. **p*<0.05; ***p*<0.01; ****p*<0.001 by Student's t test.

Supplementary Figure 4

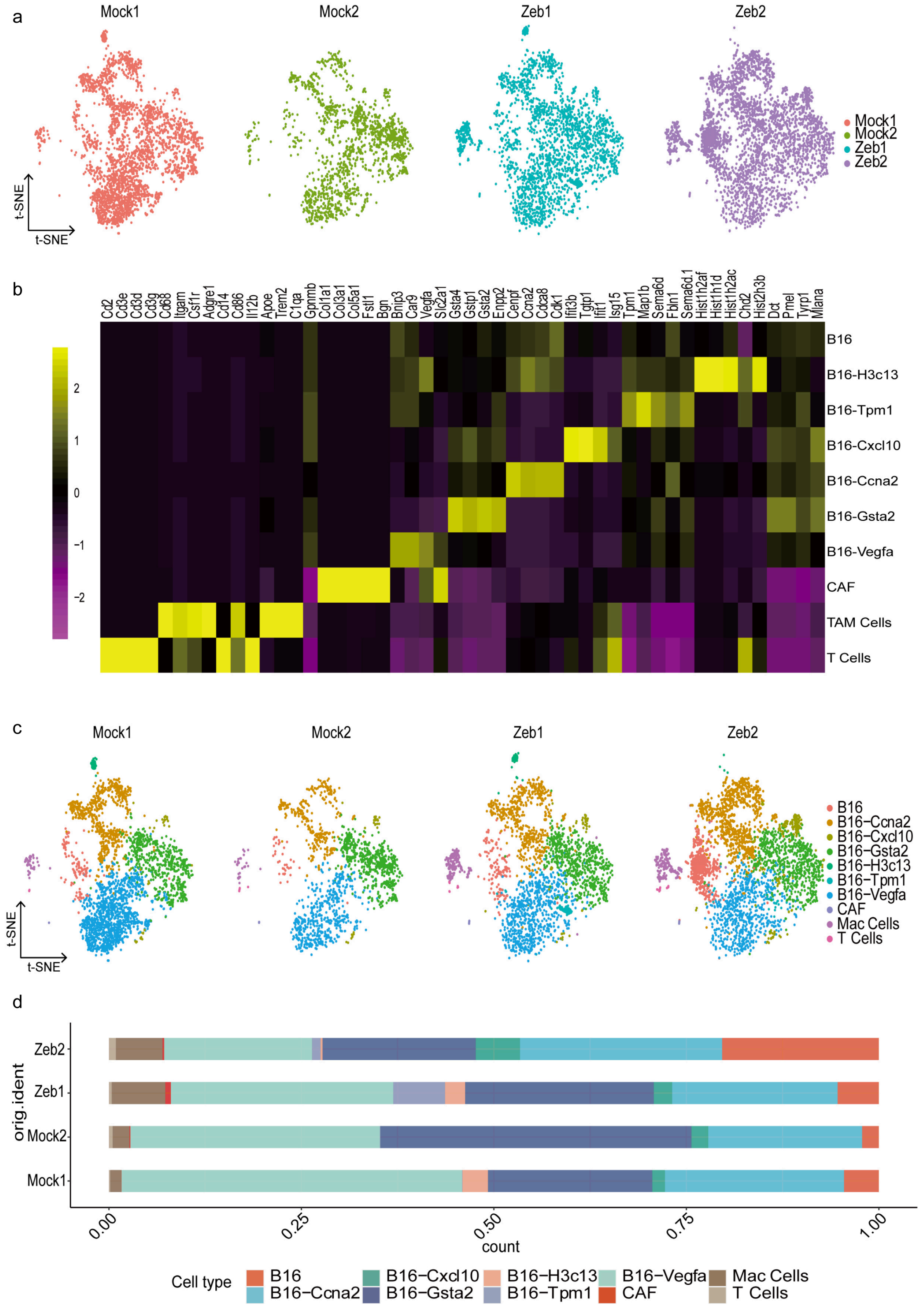

Supplementary Figure 4. Identification of intratumoral cell types by scRNA-seq.

a T-distributed Random Neighbor Embedding (t-SNE) diagram showing in mock and

zebularine group.

b Heat map showing the expression of marker genes in the indicated cell types. The color legend indicates normalized gene expression levels among the sub-clusters.

c T-distributed stochastic neighbor embedding (t-SNE) plot, showing the annotation and color codes for mock and zebularine-treated B16F10 melanoma.

d Histogram indicating the proportion of cell population clusters in tumor tissue between mock and zebularine-treated group.

Supplementary Figure 5

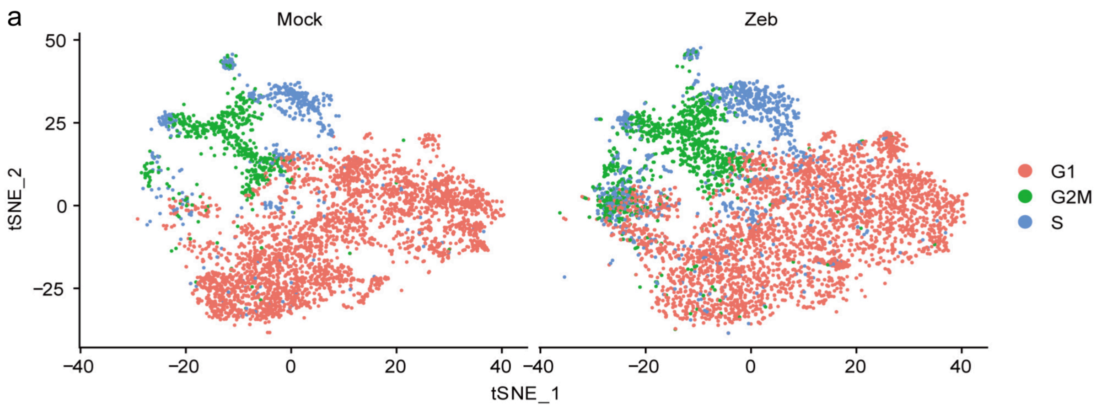

Supplementary Figure 5. Distinct functional signatures of the melanoma tumor cells

a T-distributed Random Neighbor Embedding (t-SNE) diagram showing the cell cycle after treatment of B16F10 melanoma in the mock and zebularine groups.

Supplementary Figure 6

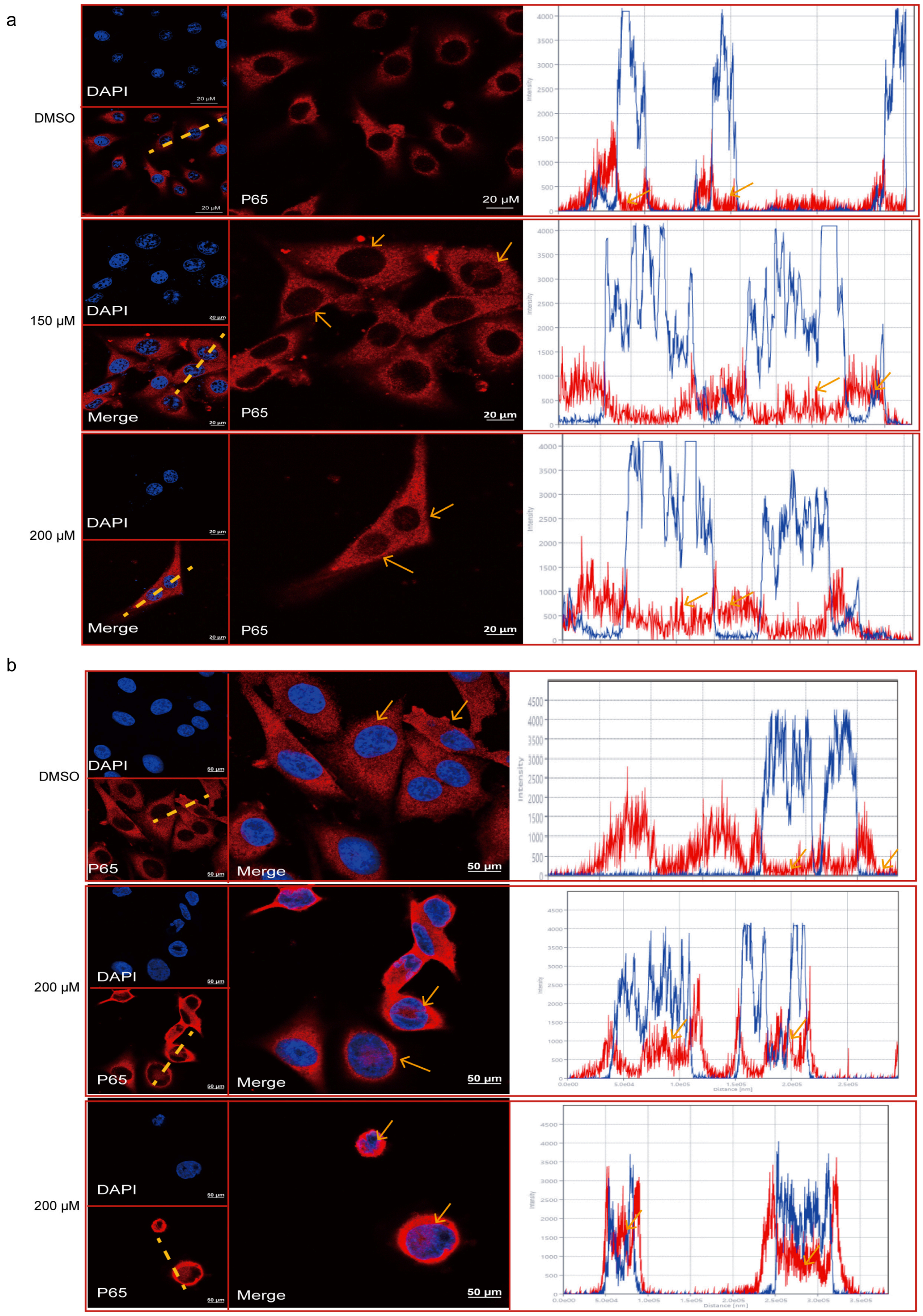

Supplementary Figure 6. Zebularine promotes NF-κB (Rela/p65) nuclear translocation.

a-b NF-κB (Rela/p65) nuclear translocation of B16F10 (a) and A375 (b) cells after treatment with different concentrations of zebularine, observed by confocal microscopy.

Supplementary Figure 7

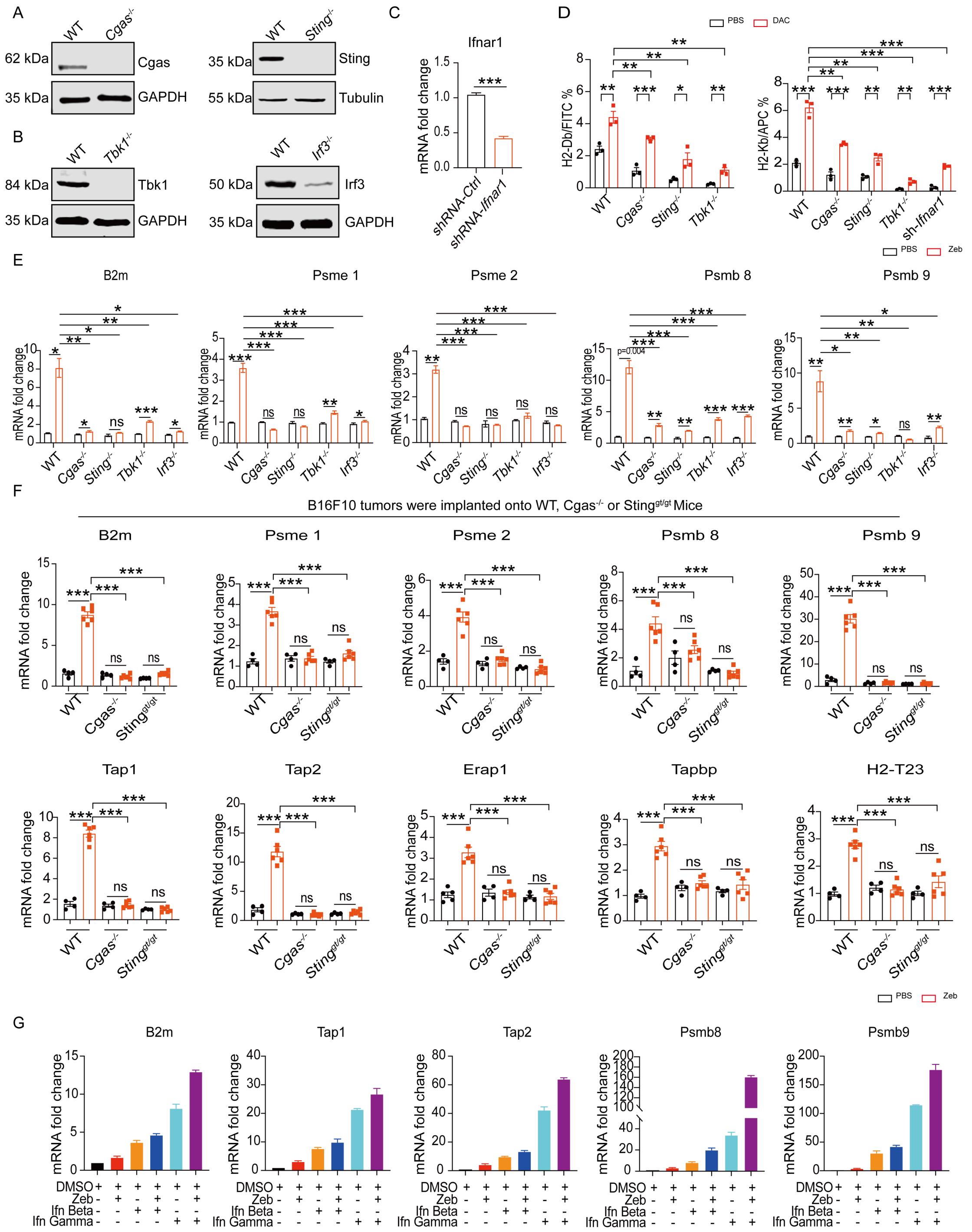

Supplementary Figure 7. Zebularine up-regulated antigen presentation and processing are dependent on cGAS-STING pathway.

a, b Immunoblot showing the effect of sgRNA-mediated *Cgas*$^{-/-}$, *Sting*$^{-/-}$, *Tbk1*$^{-/-}$, and *Irf3*$^{-/-}$ knockout in B16F10 cells (Uncropped blots show Supplementary Figure S11).

c RT-qPCR showing the effect of short hairpin RNA (shRNA)-mediated *Ifnar1* knockdown in B16F10 cells.

d CRISPR-Cas9 or shRNA-mediated gene silencing in B16F10 cells treated with decitabine (DAC) (10 μM) and analyzed for surface MHC-I (H-2Kb and H-2Db) expression by flow cytometry.

e mRNA levels of indicated genes from $Cgas^{-/-}$, $Sting^{-/-}$, $Tbk1^{-/-}$, and $Irf3^{-/-}$ knockout B16F10 cells treated with DMSO or zebularine for 72 h, measured using RT-qPCR.

f mRNA levels of indicated genes from B16F10 tumors were implanted onto wild type, $Cgas^{-/-}$, or $Sting^{gt/gt}$ mice treated with PBS or zebularine for 12 days, measured by RT-qPCR.

g mRNA levels of indicated genes from B16F10 cells treated with IFN-β or IFN -γ for 48 h, measured using RT-qPCR.

Data are presented as mean ± SEM of at least three independent experiments. *$p<0.05$; **$p<0.01$; ***$p<0.001$ by Student's t test or one-way ANOVA.

Supplementary Figure 8

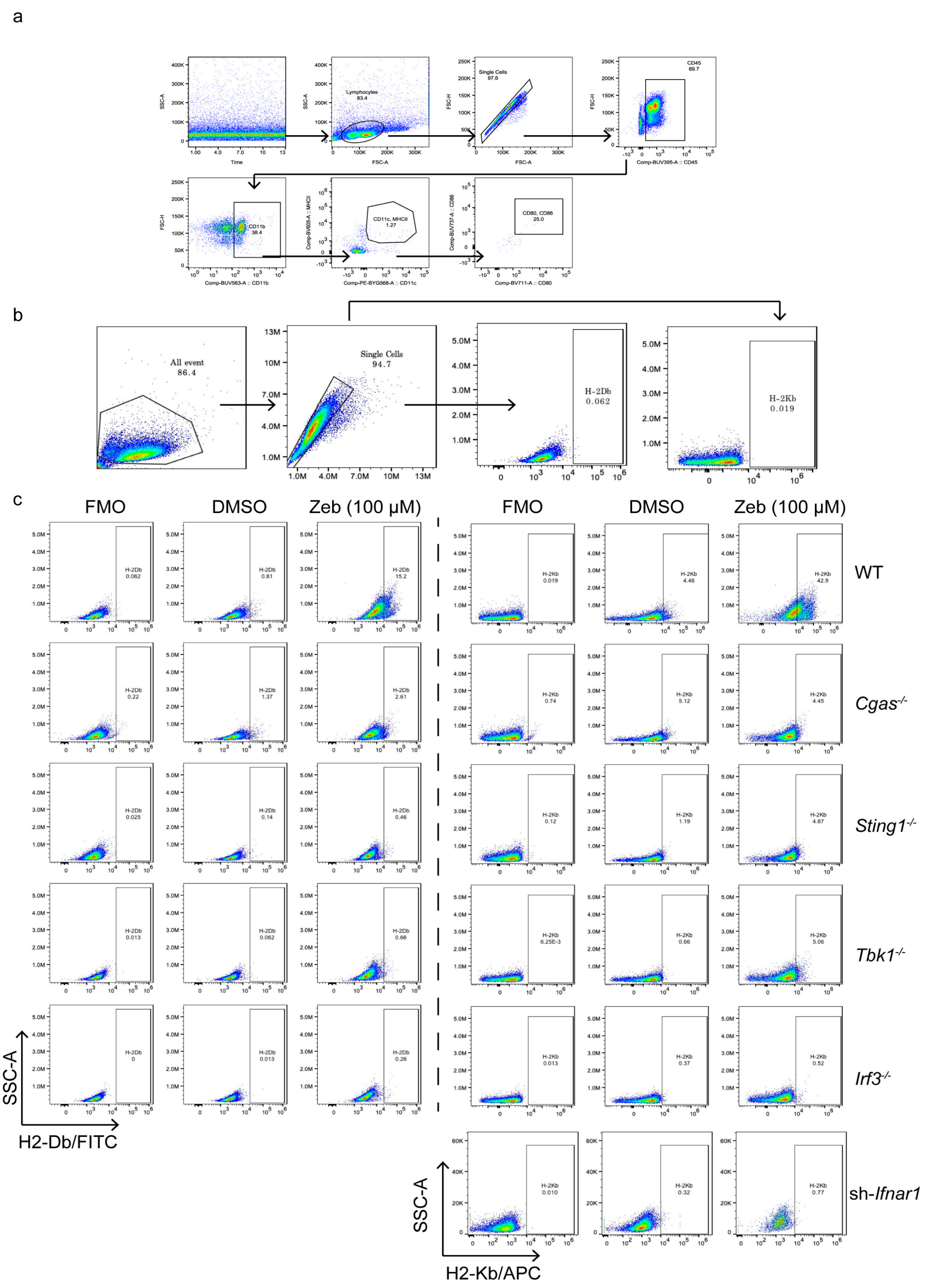

Supplementary Figure 8. Gating strategies for flow cytometry experiments.

a Gating strategy. Expression of $CD11b^+$, $CD80^+$, and $CD86^+$ cells in draining lymph nodes (dLNs) of tumor-bearing mice pre-treated with zebularine, detected by flow cytometry.

b Gating strategy. Surface expression of MHC-I (H-2Kb and H-2Db) in B16F10 cells 72 h after zebularine treatment, analyzed by flow cytometry.

c Surface expression of MHC-I (H-2Kb and H-2Db) in wild type, *Cgas*$^{-/-}$, *Sting*$^{-/-}$, *Tbk1*$^{-/-}$, *Irf3*$^{-/-}$, and sh-*Ifnar1* B16F10 cells 72 h after zebularine treatment, analyzed by flow cytometry.

Supplementary Figure 9

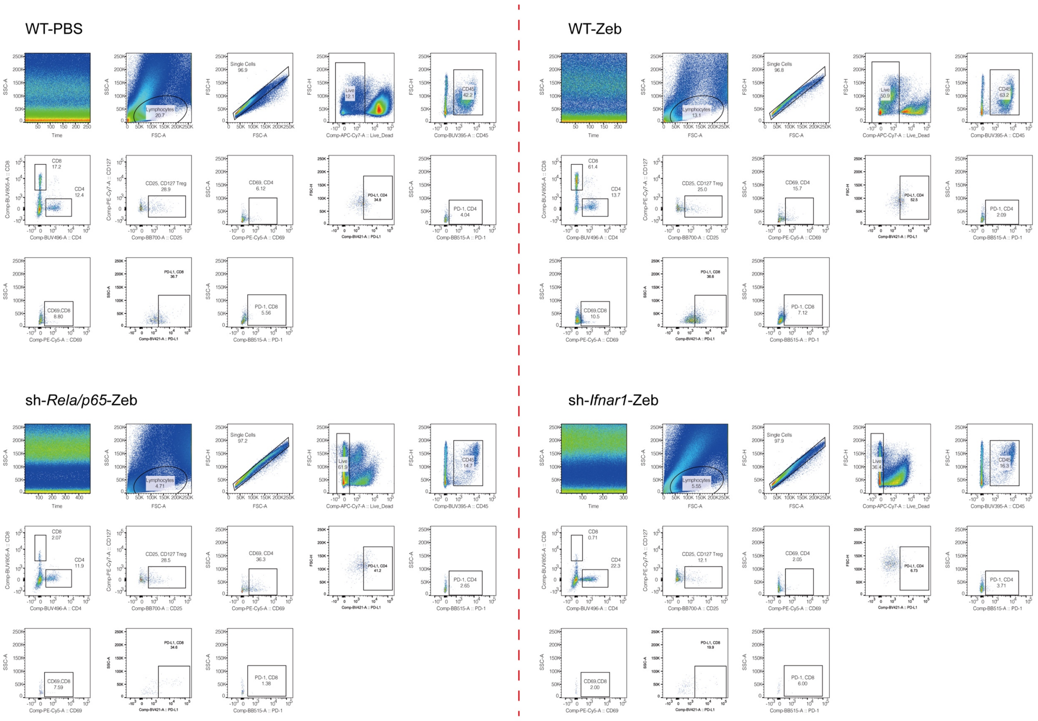

Supplementary Figure 9. Flow cytometry gating strategy for tumor-infiltrating lymphocytes (TILs) populations.

Tumor-infiltrating lymphocytes (TILs) isolated for T cell analysis by flow cytometry. Single TIL suspensions were stained for CD45, CD3, CD4, CD8, CD25, CD127, CD69, PD-1, and PD-L1. Fvs780 was used to exclude dead cells. The gating scheme for TILs is shown.

Supplementary Figure 10

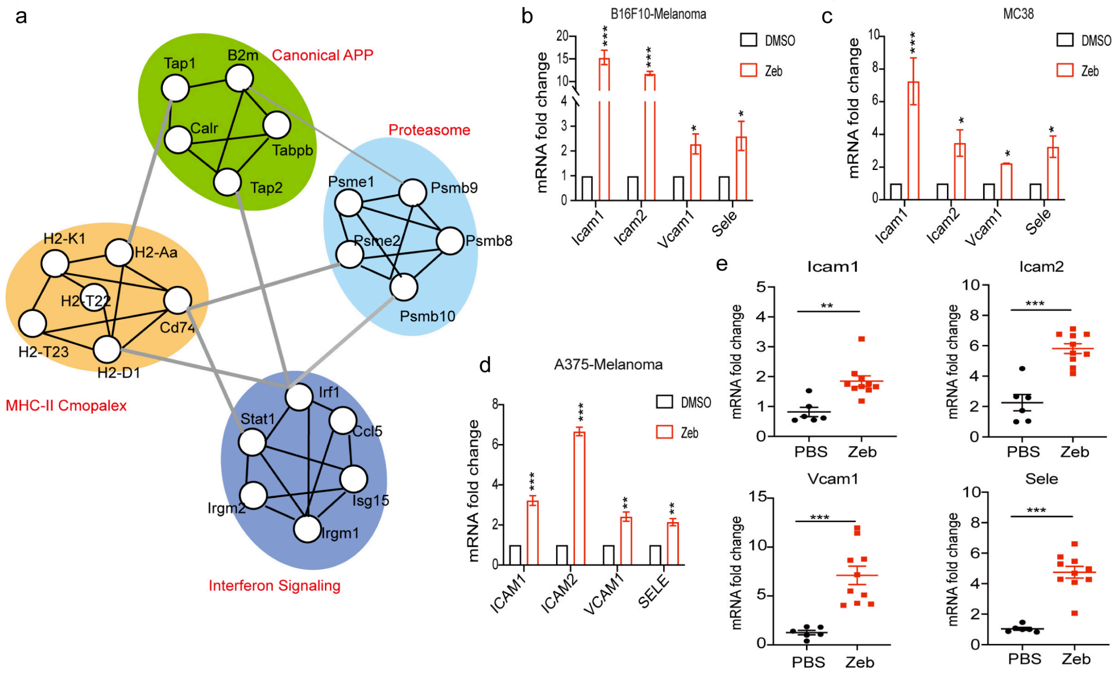

Supplementary Figure 10. Zebularine increases IFN signaling.

a Brief schematic diagram showing STRING analysis of upregulated genes.

b,c mRNA levels of indicated genes from B16F10 (b) and MC38 (c) cells treated with DMSO or zebularine for 72 h, measured using RT-qPCR.

d mRNA levels of indicated genes from A375 cells treated with DMSO or zebularine for 7 days, measured by RT-qPCR.

e mRNA levels of indicated genes from B16F10 tumor-bearing mice treated with PBS or zebularine for 12 days, measured by RT-qPCR.

Data are presented as mean ± SEM of at least three independent experiments. *p<0.05; **p<0.01; ***p<0.001 by Student's t test.

Supplementary Figure 11

Show Figure 1b

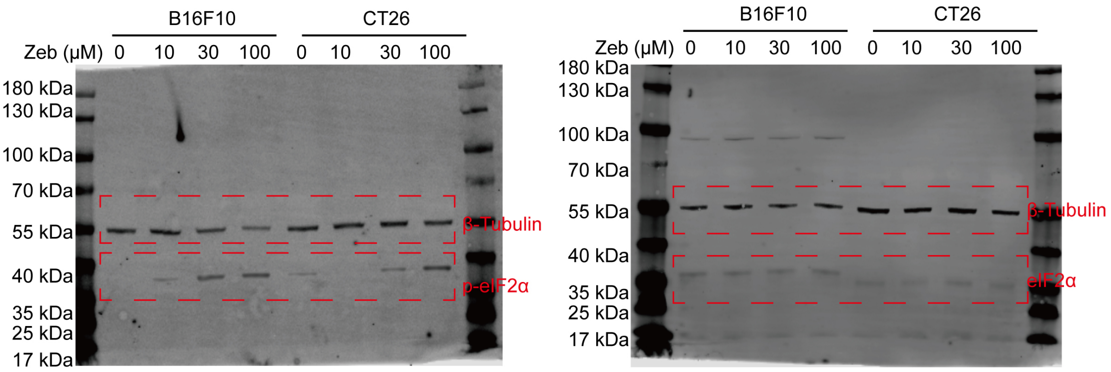

Show Figure 7a

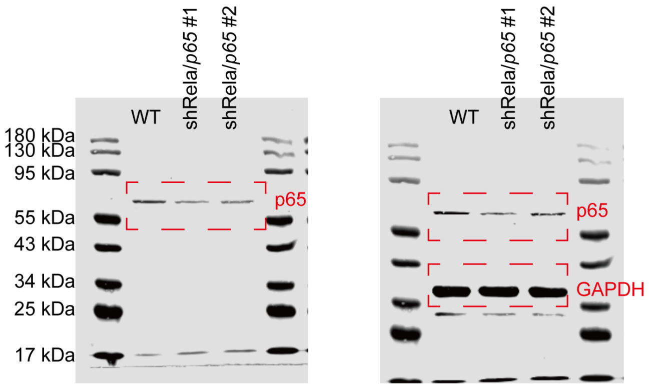

Show Figure S7a

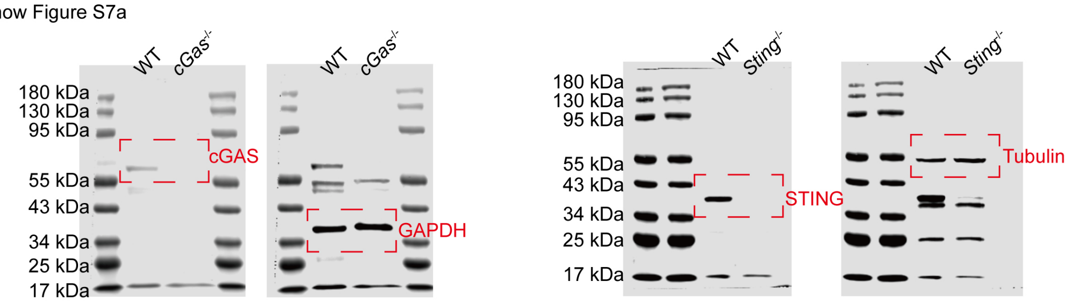

Show Figure S7b

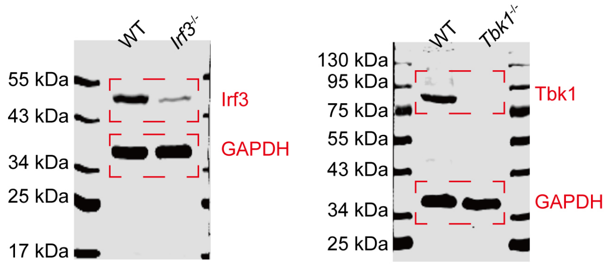

Supplementary Figure 11. Uncropped the Western blots

Supplementary Table 1

REAGENT or RESOURCE	SOURCE	IDENTIFIER
**FACS-Antibodies**		
CD16/CD32	BioLegend	
Fixable Viability Stain 780	BD Biosciences	Cat # 565388
BUV395 Rat Anti-Mouse CD45 （Clone 30-F11）	BD Biosciences	Cat # 564279
BV605 Rat Anti-Mouse I-A/I-E （Clone M5/114.15.2）	BD Biosciences	Cat # 563413
Alexa Fluor® 647 Rat Anti-Mouse F4/80 （Clone T45-2342）	BD Biosciences	Cat # 565853
BUV737 Rat Anti-Mouse CD86 （Clone GL1）	BD Biosciences	Cat # 741737
Alexa Fluor® 700 Rat Anti-Mouse CD3 Molecular Complex （Clone 17A2）	BD Biosciences	Cat # 561388
BD Horizon™ BUV496 Rat Anti-Mouse CD4（Clone GK1.5）	BD Biosciences	Cat # 612952
BV711 Hamster Anti-Mouse CD80 （Clone 16-10A1）	BD Biosciences	Cat # 740698
BUV805 Rat Anti-Mouse CD8a （Clone 53-6.7）	BD Biosciences	Cat # 612898
BUV563 Rat Anti-CD11b （Clone M1/70）	BD Biosciences	Cat # 741242
PE Hamster Anti-Mouse CD11c （Clone HL3）	BD Biosciences	Cat # 557401
PE/Cyanine5 anti-mouse CD69 Antibody (Clone H1.2F3)	BD Biosciences	Cat #104510
BD Horizon™ BB515 Rat Anti-Mouse CD279 (PD-1) (Clone RMP1-30)	BD Biosciences	Cat # 566832
BD Horizon™ BV421 Rat Anti-Mouse CD274 (Clone MIH5)	BD Biosciences	Cat # 564716
BD Horizon™ BB700 Rat Anti-Mouse CD25 (Clone PC61)	BD Biosciences	Cat # 566498
BD Pharmingen™ PE-Cy™7 Rat Anti-Mouse CD127 (Clone SB/199)	BD Biosciences	Cat # 560733
FITC anti-mouse H-2Db Antibody （Clone KH95）	BioLegend	Cat # 111505
APC anti-mouse H-2Kb Antibody (Clone AF6-88.5)	BioLegend	Cat # 116517
FITC anti-human HLA-A/B/C Antibody (Clone W6/32)	BioLegend	Cat # 311403
**Functional antibodies**		
Rat IgG2b Isotype Control – Purified in vivo PLATINUM™ Functional Grade	Leinco Technologies	Cat # R1371

Continued		
**REAGENT or RESOURCE**	**SOURCE**	**IDENTIFIER**
**Anti-Mouse CD8 (Clone YTS 169) – Purified in vivo GOLD™ Functional Grade**	Leinco Technologie s	Cat # C2442
**Anti-Mouse CD4 (Clone GK1.5) – Purified in vivo GOLD™ Functional Grade**	Leinco Technologie s	Cat # C1333
**WB-Antibodies**		
**eIF2α Rabbit mAb**	ABclonal	Cat # A21221
**Phospho-eIF2α-S51 Rabbit mAb**	ABclonal	Cat # AP0692
**Calreticulin (D3E6) XP® Rabbit mAb**	Cell Signaling Tech nology	Cat # 12238S
**HMGB1 Antibody**	Cell Signaling Tech nology	Cat # 3935
**Beta Tubulin Monoclonal antibody**	Proteintech	Cat # 66240-1-AP
**NF-κB p65 (L8F6) Mouse mAb**	Cell Signaling Tech nology	Cat # 6956
**CD81 Monoclonal Antibody**	Proteintech	Cat # 66866-1
**Donkey anti-Rabbit IgG (H+L) Highly Cross-Ad sorbed Secondary Antibody, Alexa Fluor™ 59 4**	Invitrogen	Cat # A-21207
**Donkey anti-Mouse IgG (H+L) Highly Cross-Ad sorbed Secondary Antibody, Alexa Fluor™ Plu s 488**	Invitrogen	Cat # A32766
**Chemicals, recombinant proteins**		
**Puromycin**	Gibco	A1113803
**Blasticidin**	Gibco	A1113903
**Zebularine**	Sigma-Aldrich	Cat # Z4775
**4',6'-diamidino-2-phenylindole (DAPI)**	Invitrogen	Cat # 62248
**eBioscience™ Cell Proliferation Dye eFluor™ 670**	Invitrogen	Cat # 65-0840-90
**BD Horizon™ CFSE**	BD Biosciences	Cat # 565082
**HMGB1 ELISA**	Tecan, Switzerland	Cat # ST51011
**Enhanced ATP Assay Kit**	Beyotime	Cat # S0027
**Immunostaining Permeabilization Buffer with T riton X-100**	Beyotime	Cat # P0096
**4% PFA Fix Solution**	Beyotime	Cat # P0099
**PEI Prime™ linear polyethylenimine**	Sigma-Aldrich	Cat # 919012
**Polybrene (10mg/ml)**	Solarbio	Cat # H8761
**Animal-Free Recombinant Murine IL-2**	PeproTech	Cat # AF-212-12
**Purified anti-mouse CD3ε**	Biolegend	Cat # 100302
**Purified anti-mouse CD28**	Biolegend	Cat # 102102

Continued		
**REAGENT or RESOURCE**	**SOURCE**	**IDENTIFIER**
**M-CSF Protein, Mouse, Recombinant (ECD)**	SinoBiological	Cat # 51112-MNAH
**GM-CSF Protein, Mouse, Recombinant**	SinoBiological	Cat # 51048-MNAH
**IL-4 Protein, Mouse, Recombinant**	SinoBiological	Cat # 51084-MNAE
**IFN gamma Mouse Uncoated ELISA Kit**	Thermo Fisher Scientific	Cat # 88-7314-88
**MagniSort™ Mouse T cell Enrichment Kit**	Invitrogen	Cat # 8804-6820-74
**MagniSort™ Mouse CD8 T cell Enrichment Kit**	Invitrogen	Cat # 8804-6822-74
**MagniSort™ Mouse CD4 T cell Enrichment Kit**	Invitrogen	Cat # 8804-6821-74
**Opti-MEM I Reduced Serum Medium**	Gibco	Cat # 31985070
**Enhanced ATP Assay Kit**	Beyotime	Cat # S0027
**Enhanced BCA Protein Assay Kit**	Beyotime	Cat # P0010S
**HiScript II 1st Strand cDNA Synthesis Kit (+gDNA wiper)**	Vazyme	Cat # R212
**ChamQ SYBR Color qPCR Master Mix (High ROX Premixed)**	Vazyme	Cat # Q441
**RNAiso Plus**	TaKaRa	Cat# 9108
**Critical commercial assays**		
**Qubit dsDNA BR Assay Kit**	Thermo Fisher Scie-ntific	Q32851
**Qubit™ Assay Tubes**	Thermo Fisher Scie-ntific	Q32856
**Agilent DNA High Sensitivity Kit**	Agilent Technologies	5067-4626
**AgencourtAMPureXP**	Beckman Coulter	A63880
**Single Cell 5' Library Construction Kit**	10x Genomics	Cat# 1000020
**Ms CD45 FITC (Clone 30-F11)**	BD Biosciences	Cat# 553079
**Calcein AM**	BD Biosciences	Cat # 564061
**DRAQ7**	BD Biosciences	Cat # 564904
**Stain Buffer FBS**	BD Biosciences	Cat # 554656
**Recombinant DNA**		
**lentiCRISPRv2 blast**	Addgene	Cat# 52962
**pMD2.G**	Addgene	Cat# 12259
**psPAX2**	Addgene	Cat# 12260
**pLKO.1-EGFP**	Miaolingbio	P0255

Supplementary Table 2

**Oligonucleotides**	
**Name**	**Sequence 5'- 3'**
**shRNA targeting sequence: Rela #1**	CCGGTCTGTCCTCTCACATCCGATTTCTCGAGAA<br>ATCGGATGTGAGAGGACAGTTTTTG
**shRNA targeting sequence: Rela #2**	CCGGGCTCAAGATCTGCCGAGTAAACTCGAGTTT<br>ACTCGGCAGATCTTGAGCTTTTTG
**shRNA targeting sequence: Ifnar1**	GAGTGACACCTTGCTTGTTTAT
**Crispr/Cas9 sg-Cgas-F**	CACCgCCTTACGACTTTCCGCGCCT
**Crispr/Cas9 sg-Cgas-R**	AAACAGGCGCGGAAAGTCGTAAGGc
**Crispr/Cas9 sg-Sting1-F**	CACCGGATGATCCTTTGGGTGGCAA
**Crispr/Cas9 sg-Sting1-R**	AAACTTGCCACCCAAAGGATCATCC
**Crispr/Cas9 sg-Tbk1-F**	CACCGAATCAAGAACTTATCTACGA
**Crispr/Cas9 sg-Tbk1-R**	AAACTCGTAGATAAGTTCTTGATTC
**Crispr/Cas9 sg-Irf3-F**	CACCGTGGAAGCATGGCCTACGGC
**Crispr/Cas9 sg-Irf3-R**	AAACGCCGTAGGCCATGCTTCCAC
**siRNA-Dnmt1-318-F**	GGGAGGAAAUUAAACUUACUTT
**siRNA-Dnmt1-318-R**	AGUAAGUUUAAUUUCUCCCTT
**siRNA-Dnmt1-1387-F**	GACCUGGUUUGAUACUUAUTT
**siRNA-Dnmt1-1387-R**	AUAAGUAUCAAACCAGGUCTT
**Mouse-PCR Primer**	
**Ifnar1<tm1.2Ees>-WT-F**	ACTCAGGTTCGCTCCATCAG
**Ifnar1<tm1.2Ees>-WT-R**	CTTTTAACCACTTCGCCTCGT
**Ifnar1<tm1.2Ees>-Mutant-F**	ACTCAGGTTCGCTCCATCAG
**Ifnar1<tm1.2Ees>-Mutant-R**	GAACCTGAGGCTGTCGAAGG
**Mouse-qPCR Primer**	
**m-Ifnb1-F**	TCCGAGCAGAGATCTTCAGGAA
**m-Ifnb1-R**	TGCAACCACCACTCATTCTGAG
**m-Cxcl10-F**	GCCGTCATTTTCTGCCTCA
**m-Cxcl10-R**	CGTCCTTGCGAGAGGGATC
**m-Cxcl9-F**	GAGCAGTGTGGAGTTCGAGG
**m-Cxcl9-R**	TCCGGATCTAGGCAGGTTTG
**m-Isg15-F**	CTGAAGAAGCAGATTGCCCAGAAG
**m-Isg15-R**	CGCTGCAGTTCTGTACCACTAGC
**m-Nfkb1-F**	GGAGGCATGTTCGGTAGTGG
**m-Nfkb1-R**	CCCTGCGTTGGATTTCGTG
**m-Stat1-F**	TCACAGTGGTTCGAGCTTCAG
**m-Stat1-R**	GCAAACGAGACATCATAGGCA
**m-Stat2-F**	TCCTGCCAATGGACGTTCG
**m-Stat2-R**	GTCCCACTGGTTCAGTTGGT
**m-Irf1-F**	ATGCCAATCACTCGAATGCG
**m-Irf1-R**	TTGTATCGGCCTGTGTGAATG

(Continued on next page)

Continued	
**Name**	**Sequence 5'- 3'**
**m-Irf5-F**	GGTCAACGGGGAAAAGAAACT
**m-Irf5-R**	CATCCACCCCTTCAGTGTACT
**m-Irf6-F**	CTCTCCCCATGACTGACTTGG
**m-Irf6-R**	CAGGTCCCCATAGAAGAGCC
**m-Irf7-F**	GAGACTGGCTATTGGGGGAG
**m-Irf7-R**	GACCGAAATGCTTCCAGGG
**m-Irf9-F**	GCCGAGTGGTGGGTAAGAC
**m-Irf9-R**	GCAAAGGCGCTGAACAAAGAG
**m-Ccl4-F**	TTCCTGCTGTTTCTCTTACACCT
**m-Ccl4-R**	CTGTCTGCCTCTTTTGGTCAG
**m-Ccl5-F**	GCTGCTTTGCCTACCTCTCC
**m-Ccl5-R**	TCGAGTGACAAACACGACTGC
**m-Ccl7-F**	GCTGCTTTCAGCATCCAAGTG
**m-Ccl7-R**	CCAGGGACACCGACTACTG
**m-Ccl28-F**	GTGTGTGGCTTTTCAAACCTCA
**m-Ccl28-R**	TGCATGAACTCACTCTTTCCAG
**m-Cxcl2-F**	CCAACCACCAGGCTACAGG
**m-Cxcl2-R**	GCGTCACACTCAAGCTCTG
**m-Ifi16-F**	TCAGTTTCAGTAGCCACGGTAGCA
**m-Ifi16-R**	TGGTCCCAAACAAGTGATGGTGC
**m-Ifi44-F**	CTTGGAGGATTTGCCTTTGAAC
**m-Ifi44-R**	AATCAACAGACCCAGACAAGAA
**m-Oas1f-F**	GTGAAGGATCTTAGCAGCACC
**m-Oas1f-R**	GCACCTATGAAGTCCACGTCTG
**m-Oas2-F**	GCTGGTGCTGGCATCTTCTA
**m-Oas2-R**	TTTAGATCTCTAGGTACTGGCACT
**m-Oas3-F**	TCTGGGGTCGCTAAACATCAC
**m-Oas3-R**	GATGACGAGTTCGACATCGGT
**m-B2m-F**	ACCGTCTACTGGGATCGAGA
**m-B2m-R**	TGCTATTTCTTTCTGCGTGCAT
**m-Tapbp-F**	ATACTTCAAGGTGGATGACCCG
**m-Tapbp-R**	GACTTCTAGCCCACTTCGCC
**m-Erap1-F**	GGCGGCTGTGATGAAGGTAA
**m-Erap1-R**	AGGGGAAGAAGAGAGGGCAT
**m-Tap1-F**	GAAGAACCCAGCAGGTTCCA
**m-Tap1-R**	CACCAAGAGACACAGGGCTC
**m-Tap2-F**	ACTGTGAGGACGCTCAAGTG
**m-Tap2-R**	CCCTTTTTCCCCGATTTCTGTG
**m-H2-Eb1-F**	ACGGTGTGCAGACACAACTATGAG
**m-H2-Eb1-R**	CTTGCCATTCCGGAACCATC
**m-H2-Ab1-F**	ACAGCTTATTAGGAATGGGGACT

(Continued on next page)

Continued	
**Name**	**Sequence 5'- 3'**
**m-H2-Ab1-R**	CACGGTGATGGGACTCTTCA
**m-H2-Aa-F**	TCAGTCGCAGACGGTGTTTAT
**m-H2-Aa-R**	GGGGGCTGGAATCTCAGGT
**m-H2-Q4-F**	ATGGCGTCAACAATGCTGC
**m-H2-Q4-R**	GGGACACGGAGGTGTAGAA
**m-H2-K1-F**	CGTTCCAGGGGATGTACGG
**m-H2-K1-R**	GCTCCCACTTGTGTTTGGTGA
**m-H2-T23-F**	ACAGTCCCGACCCAGAGTAG
**m-H2-T23-R**	CCACGTAGCCGACAATGATGA
**m-Cd74-F**	AGTGCGACGAGAACGGTAAC
**m-Cd74-R**	CGTTGGGGAACACACACCA
**m-Nlrc5-F**	GTGCCAAACGTCCTTTTCAGA
**m-Nlrc5-R**	AGTGAGGAGTAAGCCATGCTC
**m-Psme2-F**	GAGAAGCCCGAAAACAGGTG
**m-Psme2-R**	AGAGCTGACTCAGGGATATGATT
**m-Psme1-F**	GCCACACTGAGGGTCCATC
**m-Psme1-R**	AACGCATCCAACTCTGAGATTT
**m-Psmb8-F**	ATGGCGTTACTGGATCTGTGC
**m-Psmb8-R**	CGCGGAGAAACTGTAGTGTCC
**m-Psmb9-F**	CATGAACCGAGATGGCTCTAGT
**m-Psmb9-R**	TCATCGTAGAATTTTGGCAGCTC
**m-Icam1-F**	GTGATGCTCAGGTATCCATCCA
**m-Icam1-R**	CACAGTTCTCAAAGCACAGCG
**m-Icam2-F**	TGGTCCGAGAAGCAGATAGTAG
**m-Icam2-R**	GAGGCTGGTACACCCTGATG
**m-Vcam1-F**	AGTTGGGGATTCGGTTGTTCT
**m-Vcam1-R**	CCCCTCATTCCTTACCACCC
**m-Sele-F**	ATGCCTCGCGCTTTCTCTC
**m-Sele-R**	GTAGTCCCGCTGACAGTATGC
**m-Rela/p65-F**	AGGCTTCTGGGCCTTATGTG
**m-Rela/p65-R**	TGCTTCTCTCGCCAGGAATAC
**m-βactin-F**	ATGCTCCCCGGGCTGTAT
**m-βactin-R**	CATAGGAGTCCTTCTGACCCATTC
**m-Dnmt1-F**	AAGAATGGTGTTGTCTACCGAC
**m-Dnmt1-R**	CATCCAGGTTGCTCCCCTTG
**m-Ifnar1-F**	AGCCACGGAGAGTCAATGG
**m-Ifnar1-R**	GCTCTGACACGAAACTGTGTTTT
**m-Rela/p65-F**	AGGCTTCTGGGCCTTATGTG
**m-Rela/p65-R**	TGCTTCTCTCGCCAGGAATAC
**Human-qPCR Primer**	
**h-IFNb-F**	AGGACAGGATGAACTTTGAC

(Continued on next page)

Continued	
**Name**	**Sequence 5'- 3'**
**h-IFNb-R**	TGATAGACATTAGCCAGGAG
**h-CXCL9-F**	GTGGTGTTCTTTTCCTCTTGGG
**h-CXCL9-R**	ACAGCGACCCTTTCTCACTAC
**h-CXCL10-F**	TGGCATTCAAGGAGTACCTC
**h-CXCL10-R**	TTGTAGCAATGATCTCAACACG
**h-ISG15-F**	GAGCATCCTGGTGAGGAATAAC
**h-ISG15-R**	CGCTCACTTGCTGCTTCA
**h-B2M-F**	GAGGCTATCCAGCGTACTCCA
**h-B2M-R**	CGGCAGGCATACTCATCTTTT
**h-TAPBP-F**	TGGACCGGAAATGGGACCT
**h-TAPBP-R**	CCCCAGAAGGGTAGAAGTGG
**h-ERAP1-F**	CCCCTCAAATGGTCCCTTGC
**h-ERAP1-R**	GAGATGCTTCAGTGCTCTGAC
**h-TAP1-F**	TGCCCCGCATATTCTCCCT
**h-TAP1-R**	CACCTGCGTTTTCGCTCTTG
**h-TAP2-F**	AATCCCTCACTATTCTGGTCGT
**h-TAP2-R**	TCGAGACATGGTGTAGGTGAAG
**HLA-A-F**	ACCCTCGTCCTGCTACTCTC
**HLA-A-R**	CTGTCTCCTCGTCCCAATACT
**HLA-B-F**	CAGTTCGTGAGGTTCGACAG
**HLA-B-R**	CAGCCGTACATGCTCTGGA
**HLA-C-F**	GGACAAGAGCAGAGATACACG
**HLA-C-R**	CAAGGACAGCTAGGACAACC
**h-NLRC5-F**	AACGAGACCTTGGACCCTGAA
**h-NLRC5-R**	GCTGGTGAACCCATCATCATAG
**h-CD74-F**	GGCAACATGACAGAGGACCA
**h-CD74-R**	GCTCTCACATGGGGACTGG
**h-ICAM1-F**	ATGCCCAGACATCTGTGTCC
**h-ICAM1-R**	GGGGTCTCTATGCCCAACAA
**h-ICAM2-F**	CGGATGAGAAGGTATTCGAGGT
**h-ICAM2-R**	CACCCACTTCAGGCTGGTTAC
**h-VCAM1-F**	TTTGACAGGCTGGAGATAGACT
**h-VCAM1-R**	TCAATGTGTAATTTAGCTCGGCA
**h-SELE-F**	CAGCAAAGGTACACACACCTG
**h-SELE-R**	CAGACCCACACATTGTTGACTT
**h-βactin-F**	TCCCTGGAGAAGAGCTACG
**h-βactin-R**	GTAGTTTCGTGGATGCCACA
