# Supplementary material for: Zebularine potentiates anti-tumor immunity by inducing tumor immunogenicity and improving antigen processing through cGAS-STING pathway
Source: Commun Biol. 2024 May 16;7:587. doi: 10.1038/s42003-024-06271-w (PMC11099016; doi:10.1038/s42003-024-06271-w)
Supplement: Supplementary file 3 — Description of Additional Supplementary Files [file 42003_2024_6271_MOESM3_ESM.pdf]

Description of Additional Supplementary Files

File name: Supplementary Data
Description: The source behind the graphs in the paper.
