# Supplementary material for: Zebularine potentiates anti-tumor immunity by inducing tumor immunogenicity and improving antigen processing through cGAS-STING pathway
Source: Commun Biol. 2024 May 16;7:587. doi: 10.1038/s42003-024-06271-w (PMC11099016; doi:10.1038/s42003-024-06271-w)
Supplement: Supplementary file 5 — Reporting Summary [file 42003_2024_6271_MOESM5_ESM.pdf]

nature portfolio

Corresponding author(s): Qi Chen
Last updated by author(s): Apr 11, 2024

Reporting Summary

Nature Portfolio wishes to improve the reproducibility of the work that we publish. This form provides structure for consistency and transparency in reporting. For further information on Nature Portfolio policies, see our Editorial Policies and the Editorial Policy Checklist.

Statistics

For all statistical analyses, confirm that the following items are present in the figure legend, table legend, main text, or Methods section.

n/a	Confirmed	
☐	☒	The exact sample size (*n*) for each experimental group/condition, given as a discrete number and unit of measurement
☐	☒	A statement on whether measurements were taken from distinct samples or whether the same sample was measured repeatedly
☐	☒	The statistical test(s) used AND whether they are one- or two-sided<br>*Only common tests should be described solely by name; describe more complex techniques in the Methods section.*
☒	☐	A description of all covariates tested
☒	☐	A description of any assumptions or corrections, such as tests of normality and adjustment for multiple comparisons
☐	☒	A full description of the statistical parameters including central tendency (e.g. means) or other basic estimates (e.g. regression coefficient) AND variation (e.g. standard deviation) or associated estimates of uncertainty (e.g. confidence intervals)
☐	☒	For null hypothesis testing, the test statistic (e.g. *F*, *t*, *r*) with confidence intervals, effect sizes, degrees of freedom and *P* value noted<br>*Give P values as exact values whenever suitable.*
☒	☐	For Bayesian analysis, information on the choice of priors and Markov chain Monte Carlo settings
☒	☐	For hierarchical and complex designs, identification of the appropriate level for tests and full reporting of outcomes
☒	☐	Estimates of effect sizes (e.g. Cohen's *d*, Pearson's *r*), indicating how they were calculated

Our web collection on statistics for biologists contains articles on many of the points above.

Software and code

Policy information about availability of computer code

Data collection	Flow cytometry was conducted by BD FACSymphony A5. The western blot membranes were scanned with the ODYSSEY CLx (Gene Company). QPCR was detected using the ABI Q6 Fast Real-time PCR system (Applied Biosystems, Foster City, CA, USA). Image staining were captured with confocal microscope (LSM 780, Zeiss, Oberkochen, Germany), ELISA data were obtained from FLOUstar OPTIMA (Germany)...
Data analysis	Single-cell RNA-seq data was processed using Cell Ranger (v4.0.0) from the 10x Genomics platform, generating downstream data for subsequent analyses. We used Gene Ontology enrichment analysis and Single-sample Gene Set Enrichment Analysis (ssGSEA) for functional analysis. Gene signatures scores of samples were evaluated using R package GSVA. GO and KEGG analyses were performed by applying the "clusterProfiler" package. The western blot membranes were scanned with the ODYSSEY CLx (Gene Company) and analyzed with Image J software. Flow cytometry was conducted by Flow cytometry was conducted by BD FACSymphony A5 and analyzed with flowjo software. QPCR was detected using the ABI Q6 Fast Real-time PCR system (Applied Biosystems, Foster City, CA, USA) and analyzed with GraphPad Prism 8 software. The Schematic draw by Figdraw. Image staining was analyzed with image J software...

For manuscripts utilizing custom algorithms or software that are central to the research but not yet described in published literature, software must be made available to editors and reviewers. We strongly encourage code deposition in a community repository (e.g. GitHub). See the Nature Portfolio guidelines for submitting code & software for further information.

Data

Policy information about availability of data

All manuscripts must include a data availability statement. This statement should provide the following information, where applicable:

- Accession codes, unique identifiers, or web links for publicly available datasets
- A description of any restrictions on data availability
- For clinical datasets or third party data, please ensure that the statement adheres to our policy

The raw sequence data（Single cell RNA-Seq） reported in this paper have been deposited in the China National Center for Bioinformation, Chinese Academy of Sciences (CRA010462) that are publicly accessible at https://ngdc.cncb.ac.cn/gsa.

Research involving human participants, their data, or biological material

Policy information about studies with human participants or human data. See also policy information about sex, gender (identity/presentation), and sexual orientation and race, ethnicity and racism.

Reporting on sex and gender	N/A
Reporting on race, ethnicity, or other socially relevant groupings	N/A
Population characteristics	N/A
Recruitment	N/A
Ethics oversight	N/A

Note that full information on the approval of the study protocol must also be provided in the manuscript.

Field-specific reporting

Please select the one below that is the best fit for your research. If you are not sure, read the appropriate sections before making your selection.

☒ Life sciences ☐ Behavioural & social sciences ☐ Ecological, evolutionary & environmental sciences

For a reference copy of the document with all sections, see nature.com/documents/nr-reporting-summary-flat.pdf

Life sciences study design

All studies must disclose on these points even when the disclosure is negative.

Sample size	For mouse model and in vivo study, We have normally five mice in a group. In vitro study, we have repeated each experiment for at least three times.
Data exclusions	N/A
Replication	All experiments were conducted for 3 times and get similar results.
Randomization	All samples and mice were randomly divided into experimental groups.
Blinding	Investigators were not blinded to group during data collection and analysis because we checked tumor sizes and gave different treatments to different groups.

Reporting for specific materials, systems and methods

We require information from authors about some types of materials, experimental systems and methods used in many studies. Here, indicate whether each material, system or method listed is relevant to your study. If you are not sure if a list item applies to your research, read the appropriate section before selecting a response.

Materials & experimental systems

n/a	Involved in the study
	☒ Antibodies
	☒ Eukaryotic cell lines
☒	Palaeontology and archaeology
	☒ Animals and other organisms
☒	Clinical data
☒	Dual use research of concern
☒	Plants

Methods

n/a	Involved in the study
☒	ChIP-seq
	☒ Flow cytometry
☒	MRI-based neuroimaging

Antibodies

Antibodies used

CD16/CD32 (BioLegend)
Fixable Viability Stain 780 (BD Biosciences, Cat # 565388)
BUV395 Rat Anti-Mouse CD45 （Clone 30-F11） (BD Biosciences)
BV605 Rat Anti-Mouse I-A/I-E （Clone M5/114.15.2） (BD Biosciences)
Alexa Fluor® 647 Rat Anti-Mouse F4/80 （Clone T45-2342）
BUV737 Rat Anti-Mouse CD86 （Clone GL1） (BD Biosciences)
Alexa Fluor® 700 Rat Anti-Mouse CD3 Molecular Complex （Clone 17A2） (BD Biosciences)
BD Horizon™ BUV496 Rat Anti-Mouse CD4 （Clone GK1.5） (BD Biosciences)
BV711 Hamster Anti-Mouse CD80 （Clone 16-10A1） (BD Biosciences)
BUV805 Rat Anti-Mouse CD8a （Clone 53-6.7） (BD Biosciences)
BUV563 Rat Anti-CD11b （Clone M1/70） (BD Biosciences)
PE Hamster Anti-Mouse CD11c （Clone HL3） (BD Biosciences)
PE/Cyanine5 anti-mouse CD69 Antibody (Clone H1.2F3) (BD Biosciences)
BD Horizon™ BB515 Rat Anti-Mouse CD279 (PD-1) (Clone RMP1-30) (BD Biosciences)
BD Horizon™ BV421 Rat Anti-Mouse CD274 (Clone MIH5) (BD Biosciences)
BD Horizon™ BB700 Rat Anti-Mouse CD25 (Clone PC61) (BD Biosciences)
BD Pharmingen™ PE-Cy™7 Rat Anti-Mouse CD127 (Clone SB/199) (BD Biosciences)
FITC anti-mouse H-2Kq Antibody （Clone KH114） (BioLegend)
APC anti-mouse H-2Kd Antibody (Clone SF1-1.1) (BioLegend)
FITC anti-human HLA-A,B,C Antibody (Clone W6/32) (BioLegend)
Rat IgG2b Isotype Control – Purified in vivo PLATINUM™ Functional Grade (Leinco Technologies)
Anti-Mouse CD8 (Clone YTS 169) – Purified in vivo GOLD™ Functional Grade (Leinco Technologies)
Anti-Mouse CD4 (Clone GK1.5) – Purified in vivo GOLD™ Functional Grade (Leinco Technologies)
WB-Antibodies
eIF2α Rabbit mAb (ABclonal)
Phospho-eIF2α-S51 Rabbit mAb (ABclonal)
Calreticulin (D3E6) XP® Rabbit mAb (Cell Signaling Technology)
HMGB1 Antibody (Cell Signaling Technology)
DDIT3/CHOP Rabbit mAb (ABclonal)
BiP/GRP78 Rabbit mAb (ABclonal)
Beta Tubulin Monoclonal antibody (Proteintech)
GAPDH Monoclonal antibody (Proteintech)
NF-κB p65 (L8F6) Mouse mAb (Cell Signaling Technology)
CD81 Monoclonal Antibody (Proteintech)
Donkey anti-Rabbit IgG (H+L) Highly Cross-Adsorbed Secondary Antibody, Alexa Fluor™ 594 9 (Invitroge)
Donkey anti-Mouse IgG (H+L) Highly Cross-Adsorbed Secondary Antibody, Alexa Fluor™ Plus 488 (Invitroge)

Validation

The validation statements are available in the manufactures' websites. And their specificity was further verified by the size and location of the bands in western blot membranes.

Eukaryotic cell lines

Policy information about cell lines and Sex and Gender in Research

Cell line source(s)	HEK293T, A375, WM-266-4, B16F10, CT26, MC38, and 4T1 cells were purchased from ATCC.
Authentication	Authentication of cell lines with short tandem repeat (STR) DNA profiles was performed every 2 year with Pricella (China).
Mycoplasma contamination	All cell lines were tested negatively for mycoplasma contamination.
Commonly misidentified lines (See ICLAC register)	N/A

Animals and other research organisms

Policy information about studies involving animals; ARRIVE guidelines recommended for reporting animal research, and Sex and Gender in Research

Laboratory animals	Animal experiments were carried out in the C57BL/6J and BALB/c mouse backgrounds. Female C57BL/6 and BALB/c mice at 6-8 weeks of age were purchased from Shanghai SLAC Laboratory Animal Co. (Shanghai, China). Female NOD-SCID and NCG mice, 6-8 weeks of age, were purchased from GemPharmatech Company (Jiangsu, China). B6(C)-Cgastm1d(EUCOMM)Hmgu/J mice (Strain: 026554), C57BL/6J-Tmem173gt/J mice (Strain: 017537) and B6(Cg)-Ifnar1tm1.2Ees/J mice (Strain: 028288) were purchased from The Jackson Laboratory (Bar Harbor, ME, USA). Mice were maintained in a Specific Pathogen-Free (SPF) animal facility at 23-25°C and 50-60% humidity, and with 12 hr light/12 hr dark cycles.
Wild animals	N/A
Reporting on sex	In immunological experiments, female mice are usually selected because androgen level of male mice can affect experimental results.
Field-collected samples	N/A
Ethics oversight	All experiments were conducted in accordance with the Guide for the Care and Use of Laboratory Animals approved by the Fujian Provincial Office for Managing Laboratory Animals and was guided by the Fujian Normal University Animal Care and Use Committee (Approval No. IACUC-20190004).

Note that full information on the approval of the study protocol must also be provided in the manuscript.

Plants

Seed stocks	N/A
Novel plant genotypes	N/A
Authentication	N/A

Flow Cytometry

Plots

Confirm that:

☒ The axis labels state the marker and fluorochrome used (e.g. CD4-FITC).

☒ The axis scales are clearly visible. Include numbers along axes only for bottom left plot of group (a 'group' is an analysis of identical markers).

☒ All plots are contour plots with outliers or pseudocolor plots.

☒ A numerical value for number of cells or percentage (with statistics) is provided.

Methodology

Sample preparation	Mouse lymph nodes and tumor tissues were dissociated and filtered through 70-µm strainers. Cells were incubated with Fixable Viability Stain 780 (Fvs780) for 15 min in the dark (room temperature), washed 2× with cold PBS (2% fetal bovine serum), and the Fc receptors on the cell surface were blocked with anti-CD16/32 antibody (10 µg/mL) for 15 min in the dark (4°C). Then the cells were stained with fluorophore-labeled antibodies for 30 min in the dark (4°C) and then detected by flow cytometry.
Instrument	BD FACSymphony A5
Software	Data were acquired with a BD FACSymphony A5 using FACS-Diva 7 software following the standard gating strategy for flow cytometry analysis. The data was processed using FlowJo V10.6 software.
Cell population abundance	We removed red cells withred cell ysis buffer to ensure that the cells for flow cytometry were tumor cells and lymphocytes . And we gate lymphocytes according to the FSC and SSC value.
Gating strategy	Gate cells exclude dead cells and tumor cells based on cell size, then gate Fvs780 negative cells as live cells. Then gate CD45+

Gating strategy

cells and gate CD45+CD3+ cells, then gate CD45+CD3+CD8+ as CD8+ T cells and CD45+CD3+CD4+ as CD4+ T cells. For DCs cells gating, we gate CD45+CD11c+ CD80+CD86+ cells as DCs......

☒ Tick this box to confirm that a figure exemplifying the gating strategy is provided in the Supplementary Information.
